# Effect of vibration on pain during subcutaneous heparin injection: A randomized, single-blind, placebo-controlled trial

**DOI:** 10.1515/med-2025-1272

**Published:** 2025-11-04

**Authors:** Dilek Yılmaz, Bahar Gülşah Güney

**Affiliations:** Department of Nursing, Faculty of Health Sciences, Bursa Uludağ University, 16059, Nilüfer/Bursa, Turkey; Department of Neonatal Intensive Care Unit, Ankara Bilkent City Hospital-Maternity Hospital, 06800, Çankaya/Ankara, Turkey

**Keywords:** nursing, subcutaneous, heparin, pain, vibration

## Abstract

**Objectives:**

The administration of subcutaneous low molecular weight heparin (LMWH) injections frequently causes injection pain, disrupting the comfort of patients. No studies were found evaluating the effect of the application of local vibration in the management of pain relating to subcutaneous LMWH injection. The aim of this study is to examine the effect on injection pain of the local vibration technique applied to the injection site during subcutaneous LMWH injection.

**Methods:**

The patients were randomly assigned to an experimental (vibration) group (*n* = 32), a placebo control group (*n* = 30), and a nonintervention control group (*n* = 31). Participants in the experimental group were given slight vibration to the injection site before the injection was administered; for participants in the placebo group, the device was placed on the injection site but with the vibration button kept switched off, while for the nonintervention control group, routine subcutaneous LMWH injection was administered. The level of pain felt by the participants during the administration of the injection was assessed with a visual analog scale.

**Results:**

The pain score of the participants during the subcutaneous LMWH injection was found to be significantly lower in the vibration group than in the control group (*p* < 0.001) and the placebo control group (*p* = 0.005), but there was no significant difference between the control and placebo control groups (*p* = 0.435).

**Conclusion:**

It was found that the local vibration technique applied to the site of subcutaneous LMWH injection was effective in reducing the pain developing in relation to the injection. Healthcare professionals can use the vibration technique in the management of the pain relating to subcutaneous heparin injection.

## 1 Introduction

Injections are the method of choice for administering drugs so as to achieve the desired effect directly and rapidly [1]. Subcutaneous injections are a major part of drug administrations, and they are frequently used in clinical practice [2,3]. In subcutaneous injections, the drug is injected into loose connective tissue under the skin where there are fewer blood vessels [4]. Subcutaneous injection is a parenteral drug administration route, and is generally used to administer drugs such as vaccines, insulin, hormones, and low molecular weight heparin (LMWH) [5].

Subcutaneous LMWH obtained from the fractionation of heparin is usually administrated to the subcutaneous tissue [4]. LMWH is prepared by depolymerization of the common form of heparin [6]. Subcutaneous heparin treatment is one of the essential treatment methods which is of vital importance in clinics [3]. Subcutaneous LMWH injection is administered to patients at risk of developing thromboembolism or to those who have thromboembolic disease [7]. It has the advantages of high bioavailability, a strong antithrombotic effect, and fewer bleeding side effects [6]. Therefore, patients generally receive subcutaneous LMWH for a few days or weeks from the time they are admitted to hospital until after they are discharged [8].

Systemic and local complications may occur due to subcutaneous LMWH injections. The most important complications that occur locally are pain at the injection site, ecchymosis, and hematoma [9,10,11]. Also, pain, a frequent complication complained by patients in subcutaneous heparin injection, results from the presence of pain receptors in the subcutaneous tissue [2,10] and the tissue damage caused by the LMWH solution [12,13]. This can cause anxiety, loss of comfort, restriction at the injection site, and refusal of treatment in patients [7,9]. In order to prevent or reduce this, it is important to apply suitable techniques when administering subcutaneous LMWH injections [12].

### 1.1 Theoretical background

Non-pharmacological methods are frequently used to manage the pain of subcutaneous heparin injections [14]. These are one complementary element in the comprehensive approach to pain reduction. Methods used in non-pharmacological treatments mostly affect the emotional, cognitive, behavioral, and sociocultural aspects of pain [15,16]. There have been studies evaluating a considerable number of non-pharmacological methods to manage the pain of subcutaneous LMWH injections. Examining these studies, it is seen that they recommend such techniques as applying cold to the injection site [6], applying manual pressure to the injection site [3,17], using the ShotBlocker device at the injection site [1], extending the duration of the injection [18], cryotherapy [1], or using the Valsalva maneuver [14].

One of the non-pharmacological methods of pain control, the vibration technique, has recently started to be used. It has been reported in studies that the local vibration technique is effective in controlling the pain of invasive procedures [19,20,21]. It is explained by the gate control theory [22]. This theory suggests that pain is transmitted from the peripheral nervous system to the central nervous system. It has been suggested that the afferent pain-receptive nerves are blocked by faster non-noxious motion nerves [23]. Despite evaluations of the effect of the local vibration technique administered by itself without combining it with any other technique on the pain of the Pfizer–BioNTech COVID-19 vaccination [22], intramuscular antibiotic injection [19] and intramuscular injection of ceftriaxone [20], blood glucose measurement [24], and insulin injection [25], no studies were found which evaluated its effect on controlling the pain of subcutaneous heparin injection.

Nurses have an important role in minimizing the degree of discomfort sensation and pain during any invasive procedure, together with ensuring the appropriate preparation and administration of drugs [1]. When giving subcutaneous heparin injections, nurses must use techniques, which will reduce the pain in order to increase satisfaction with the quality of nursing which patients receive, to increase patient trust, and to encourage patients to cooperate with treatment [6]. It was seen that in chest diseases clinics, where subcutaneous LMWH injections are very frequently given, nurses were mostly undecided about which method to use to control injection pain. Nurses should use a technique that is cheap, effective, and easy to use in reducing the pain of subcutaneous LMWH injections and increasing patient comfort. It is remarkable that even though the local vibration technique has been recently shown to be effective in controlling the pain of invasive procedures [19,20,21,22], the effectiveness of this technique in the administration of subcutaneous LMWH injections has not been investigated. As there were very few results of relevant studies, a need was felt for this study to be performed. It is felt that the results of the study will fill a gap in the literature on the management of the pain of subcutaneous LMWH injections. The aim of this study is to examine the effect on injection pain of the local vibration technique applied to the injection site during subcutaneous LMWH injection.

The hypotheses of this research are as follows:

**Hypothesis 0.** The local vibration technique, applied to the injection site during subcutaneous LMWH injection, does not affect the intensity of injection-related pain.

**Hypothesis 1.** The local vibration technique, applied to the injection site during subcutaneous LMWH injection, reduces the intensity of injection-related pain.

## 2 Materials and methods

### 2.1 Study design

**Figure 1:**
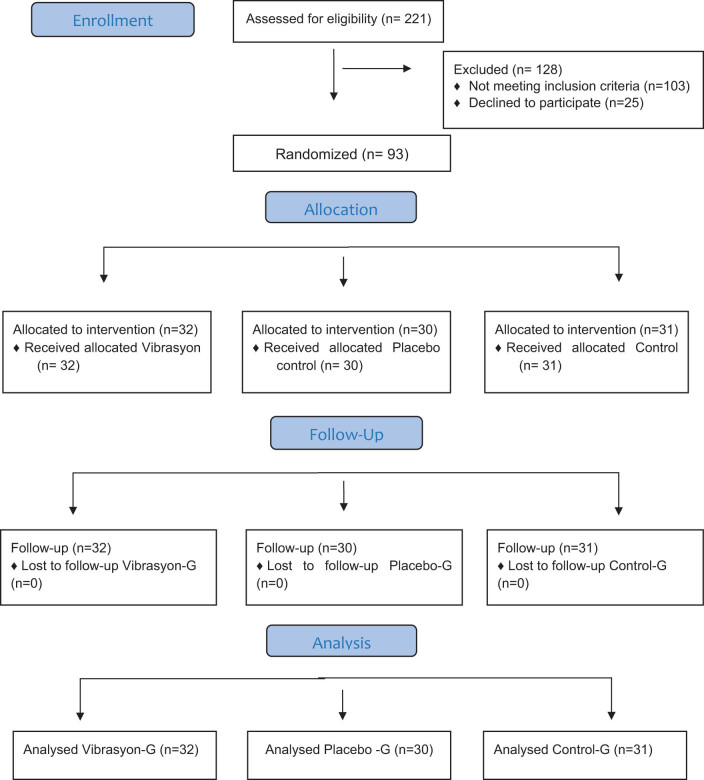
Flow chart of the Consolidated Standards of Reporting Trials shows the number of the participants through each stage of the study.

The research was conducted as a randomized, single-blinded, placebo-controlled experimental study. The flow diagram of the stages in the study procedure is given in Figure 1.

### 2.2 Setting

The research was conducted between December 2023 and May 2024 at the Chest Diseases Clinic of a university hospital in Turkey. The hospital where the research was conducted is a research hospital with a capacity of 900 beds, and a total of approximately 1,500 academic, administrative and health personnel. It provides outpatient services to an average of 300–3,500 patients a day, with modern clinic and polyclinic facilities. The Chest Diseases Clinic where the research was conducted has 17 beds. The patients generally had medical diagnoses and were under inpatient treatment in the clinic for chronic obstructive pulmonary disease, asthma, pneumonia, or bronchiectasis. At the end of the clinic there is a three-bed intensive care unit and a bronchoscopy unit. Subcutaneous LMWH injections are frequently given by the nurses in the clinic.

### 2.3 Participants and sampling

On the dates when the research was conducted, the population consisted of 221 persons. The sample size was determined using G*Power based on a significance level (*α*) of 0.05, a power level of 80 and an effective size of 80. In this way, the total sample size was determined as 84 participants, and taking account of the drop-out rate of each group, the final total number of participants was set at 93. The number of participants included in the vibration group was 32, with 30 in the placebo control group and 30 in the nonintervention control group.

Criteria for inclusion in the research were being over the age of 18, having a doctor’s prescription for subcutaneous LMWH 0.6 mL treatment with a ready-to-use syringe and not yet having begun treatment, having no coagulation disorder, having no disorder which could affect pain perception, having no incision, lipodystrophy or finding of infection at the injection site, having no communication problem, and voluntarily agreeing to participate in the research. Criteria for exclusion from the research were determined as being under the age of 18, having diabetes mellitus, peripheral vascular disease, etc., which could affect the perception of pain, having received analgesic treatment within the previous 6 h, not being conscious, refusing to participate in the research, or opting to leave the study at any point.

### 2.4 Randomization

A simple and stratified randomization method was used in the study. The aim of stratified randomization is that some studies state that age [20,26] and gender [27] have an effect on pain. For this reason, in order to check the effect of age and gender on the results of the study interventions and thereby increase the reliability of the results, individuals were classified according to age and gender when randomization was performed. Those who agreed to participate in the research were assigned to a group by means of an electronically generated list of random numbers from one to three. Those with the number 1 were assigned to the vibration group, those with the number 2 to the placebo control group, and those with the number 3 to the nonintervention control group. The main author was not involved in the randomization process, the assessments, or the interventions.

### 2.5 Outcomes of the study

The primary outcome of this study was the examination of the effect on injection pain of the local vibration technique applied to the injection site during subcutaneous LMWH injection.

The secondary outcome was the determination of the effects of the variables of age, gender, and body mass index (BMI), which could affect the intensity of pain developing as a result of subcutaneous LMWH injection in these individuals.

### 2.6 Data collection instruments

#### 2.6.1 Demographic questionnaire

This form included information on the participant’s age, gender, height, weight, and BMI.

#### 2.6.2 Visual analog scale (VAS)

A 10 cm vertical VAS was used to evaluate the severity of pain felt by the participants during the procedure. One end indicated lack of pain and the other the most severe pain possible [28]. Pain severity was evaluated in millimeters.

### 2.7 Intervention

After the voluntary participation of the patients included in the research had been secured, their descriptive characteristics were collected from the demographic questionnaire. After that, they were given information on the use of the VAS.

All injections were given to each patient once a day, in the morning at 11.30. All injections were performed by the same researcher. It is reported in the literature that because muscle activity is low, it is rich in fatty tissue, and it is wide enough to allow rotation, the area of first choice for subcutaneous injections must be the abdominal region [8,29]. Therefore, all of the subcutaneous injections were performed in the right lower abdominal region outside an area within 5 cm of the navel. As some studies report that factors such as needle length, needle diameter, or injection duration may affect injection pain [12,13], a standard injection protocol was applied to all groups in the research in order to achieve standardization.

After each injection, another researcher, who had no prior knowledge of which technique was used, immediately assessed pain intensities using the VAS and recorded them on the data collection form with the numerical value equivalent of the point, which the patient had marked. This researcher was not with the patient when the injection was given, and during treatment, the patient’s room was kept closed. Immediately after the injection was given, the researcher giving the injection left the patient’s room, and the other researcher entered the room in order to evaluate the patient’s pain intensity. After each injection, the patient’s pain severity was assessed by the same researcher.

### 2.8 Protocol of experimental and control groups

#### 2.8.1 Vibration (experimental group)

**Figure 2:**
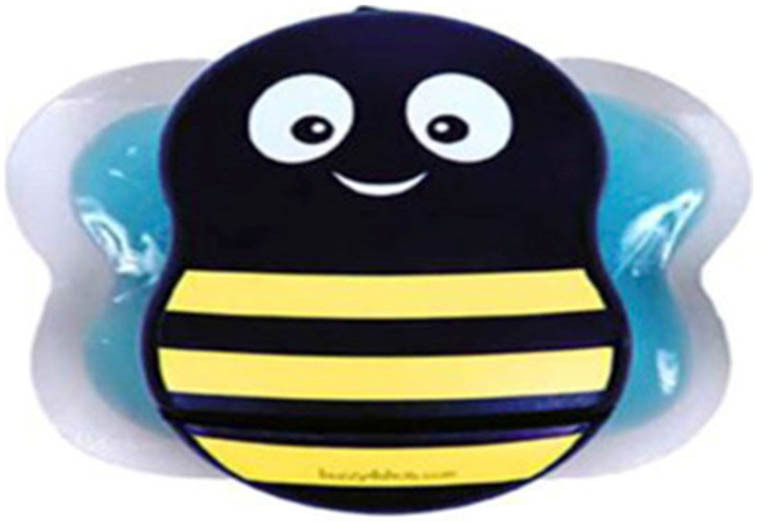
Buzzy^®^. (*Reference; Buzzy* ^®^ *- PainCareLabs*. https://paincarelabs.com).

The Buzzy^®^ device was used to provide vibration to the participants in this group. This device, designed as a combination of cold application and local vibration to reduce pain experienced during invasive procedures, is frequently chosen for all age groups [30]. Buzzy^®^ is a reusable vibrating plastic bee with an attached ice pack (Figure 2). There is a motor in the body which provides vibration. The device also has ice wings behind the body, which are used to provide cold therapy, but these were not used in this research.

In this study, only the body of the Buzzy^®^ device was used to provide vibration. The Buzzy^®^ device, at room temperature, was placed by the researcher 5 cm proximal to the injection site before the injection was performed. One minute before the injection was given, the Buzzy^®^ device was started. After that, the needle was inserted in the tissue for the injection, and while the injection was being given, vibration was continued. As Buzzy^®^ is reusable, it was disinfected after each use, before being used on the next participant.

#### 2.8.2 Placebo (intervention control group)

The Buzzy^®^ device was used with participants in this group. The device, at room temperature, was placed by the researcher 5 cm proximal to the injection site before the injection was performed. With participants in this group, the device was placed on the procedure site, but no vibration was made. The device was held on the participants for 1 min before the injection and throughout the procedure, with the vibration switched off.

#### 2.8.3 Control (nonintervention group)

No intervention was performed before or during the procedure with the participants in the control group, and the standard subcutaneous LMWH injection procedure was carried out.

### 2.9 Data analysis

Statistical analyses were performed with IBM SPSS ver. 28.0 (IBM Corp. Released 2021. IBM SPSS Statistics for Windows, Version 28.0. Armonk, NY: IBM Corp.) The data were examined by the Shapiro–Wilk test to determine whether or not it presented normal distribution. The descriptive statistics were presented as mean value ± standard deviation, frequency, and percentage. The Kruskal-Wallis and Mann-Whitney U tests were used in the comparisons of independent groups. The Bonferroni test was used as a multiple comparison test. Categorical variables were compared using Pearson’s chi-square test between groups. The significance level was taken as *p* < 0.05.


**Ethical considerations:** The study was performed in accordance with the principles of the Declaration of Helsinki. It was approved by the Institutional Review Board of Bursa Uludag University (IRB approval number 2023-28/1) and registered in the ClinicalTrials.gov Protocol Registration and Results System (https://clinicaltrials.gov/) with trial registration number NCT06469112. Participants were given clear information regarding the study, and written statements of voluntary participation were obtained before the procedures were implemented.

## 3 Results

### 3.1 Participants

The mean age of all those participating in the research was 63.07 years (SD = 14.76), and their mean BMI was 26.08 kg/m^2^ (SD = 6.26). It was found that 53.8% of the participants were male. Homogeneity was identified within all groups of the 93 participants in the study (Table 1).

**Table 1:** Comparison of participants’ variables in the groups (*n* = 93)

Variable	Categories	*n* (%) or mean value ± SD					
		Vibration	Placebo	Control	Total	Test value	*p*
		(*n* = 32)	(*n* = 30)	(*n* = 31)			
Gender^a^	Female	12 (37.5)	17 (56.7)	14 (45.2)	43 (46.2)	2.310	0.315
	Male	20 (62.5)	13 (43.3)	17 (54.8)	50 (53.8)		
Age (years)^b^		62.15 ± 15.09	64.70 ± 11.71	62.45 ± 17.21	63.07 ± 14.76	0.666	0.717
BMI^b^		26.47 ± 6.28	27.02 ± 7.09	24.75 ± 5.28	26.08 ± 6.26	2.788	0.248

Abbreviations: SD, standard deviation; BMI, body mass index. ^a^Pearson’s Chi-square test. ^b^Kruskal-Wallis test.

### 3.2 Groups’ VAS scores

**Figure 3:**
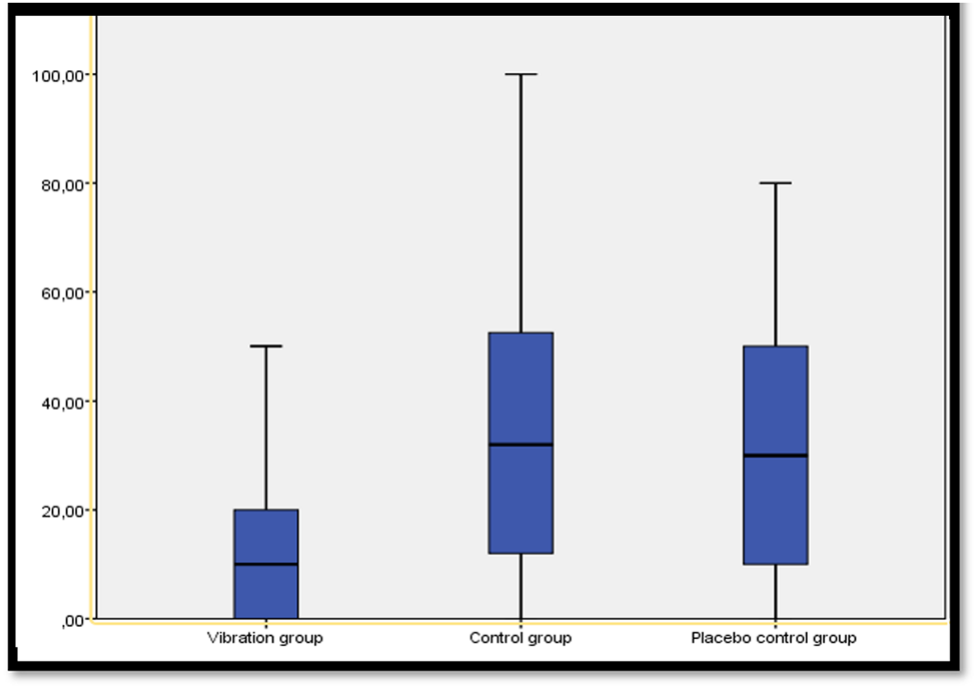
Comparison of groups’ perceived pain severity (*n* = 93).

It was found that the pain scores relating to the subcutaneous LMWH injection of the participants in the experimental and control groups were, in order, 12.46 (SD = 13.20) in the vibration group, 29.53 (SD = 21.70) in the placebo group, and 37.32 (SD = 25.47) in the control group (Figure 3). Results of the statistical analysis showed a statistically significant difference between the mean pain scores of individuals in the groups (*χ* ^2^ = 20.11, *p* < 0,001). It was found as a result of *post hoc* Bonferroni adjustments of the VAS score differences between the groups that VAS was significantly lower in the vibration group compared with the control group (*p* < 0.001) and the placebo control group (*p* = 0.005), but that there was no significant effect between the control and placebo control groups (*p* = 0.435) (Table 2).

**Table 2:** Comparison of the differences between the experimental and control groups (*n* = 93)

Variables			Difference		95% confidence interval	
	Comparison between groups		Mean value ± SD	*p*	Lower bound	Upper bound
VAS	Control	Vibration	24.85 ± 5.21^a^	0.000	12.13	37.57
		Placebo	7.78 ± 5.29	0.435	−5.14	20.71

Abbreviations: SD, standard deviation; VAS, visual analog scale. ^a^By *post hoc* Bonferroni test, *p* < 0.05.

### 3.3 Participants’ pain severity by age, gender, and BMI

Table 3 shows the pain intensity score means of all participants included in the study according to their gender, age, and BMI. According to the results of the statistical analysis, it was found that the variables of gender, age, and BMI did not affect the intensity means of the pain developing during subcutaneous LMWH injection (*p* = 0.492, *p* = 0.181, *p* = 0.497, respectively, Table 3).

**Table 3:** Comparison of participants’ pain severity by age, gender, and BMI (*n* = 93)

Variable	Categories	Mean value ± SD			
		*n* (%)	VAS	Test value	*p*
Age^a^	19–65 age group	44 (47.3)	23.54 ± 24.11	905.50	0.181
	66 or older	49 (52.7)	28.69 ± 21.94		
Gender^a^	Female	43 (46.2)	28.41 ± 24.24	986.50	0.492
	Male	50 (53.8)	24.40 ± 21.98		
BMI^b^	Underweight	9 (9.7)	20.11 ± 17.32	2.381	0.497
	Normal	37 (39.8)	28.62 ± 20.13		
	Overweight	26 (28.0)	25.07 ± 27.94		
	Obese	21 (22.6)	26.19 ± 24.12		

Abbreviations: SD, standard deviation; BMI, body mass index; VAS, visual analog scale. ^a^Mann Whitney U test. ^b^Kruskal-Wallis test.

## 4 Discussion

One of the most widespread complications occurring in connection with subcutaneous LMWH injections is pain [14,29]. Nerve endings in the subcutaneous tissue may cause a greater feeling of pain when the patient is given an injection [12]. Poor management methods of repeated subcutaneous LMWH injections can cause greater pain and anxiety [1]. Also, the pain experienced in subcutaneous heparin injections can have a negative effect on an individual’s comfort [17]. One of the basic responsibilities of nurses is to use the correct techniques to protect patients from avoidable side effects [4]. The American Society for Pain Management Nursing endorses pain control before and during painful procedures [31]. Pain management is one of the basic elements of nursing care, and nurses take on important responsibilities in this regard [32]. The quality of pain management depends on the nurse’s knowledge, attitude, and skill regarding painful interventions [19].

This study was conducted with the aim of investigating the effect on injection pain of local vibration used during subcutaneous LMWH injection. It was found as a result of the study that the application of local vibration was effective in reducing the pain of subcutaneous LMWH injection. Thus, Hypothesis H0 was rejected and Hypothesis H1 was accepted. It is thought that the results of the study will help to reduce the pain arising from the administration of subcutaneous LMWH injections and to increase patient comfort. However, since our study was performed with a small group, the need to repeat the work with larger groups should be considered. This may contribute to strengthening the study results.

An examination of the literature showed no studies evaluating the effect of the application of local vibration during subcutaneous LMWH injections. It was seen that the effect of the vibration technique on pain was assessed with various other invasive procedures. In a study by Yılmaz et al. [22], an investigation was made of the effect on injection pain in Pfizer-BioNTech COVID-19 vaccination of the methods of vibration applied to the injection site and having the individuals squeeze a stress ball. It was concluded as a result of the study that the local vibration technique was more effective in reducing pain. It was reported that vibration used during intramuscular injections was the most effective technique in reducing pain [19,20]. In a study, it was found that there was some evidence that the application of vibration was effective in reducing the pain of invasive procedures in children [21]. In a study conducted with children admitted to the emergency service, it was found that vibration intervention administered to children during intramuscular injection reduced pain and anxiety, according to the evaluations of the mothers and nurses [20]. In a meta-analysis conducted on the subject, it was reported that the application of vibration in the pediatric population during local anesthesia injections and venipuncture, and in adults with botox, intramuscular antibiotic and intralesional cortisone injections, could help to minimize the discomfort of the injections [23]. Vejdanihemmat et al. [24] conducted a study to evaluate the effect on patients’ pain perception of the local application of cold and vibration during the insertion of a glucometer needle in diabetic patients. It was concluded as a result of this study that the vibration technique was the most effective method of reducing the pain relating to the insertion of the glucometer needle. It was recommended in the study that this technique should be used by clinicians when monitoring glucose, in order to improve patient comfort. Similarly, in a study by Abdulyemmah and Majeed [25], it was stated that the local vibration technique was an effective non-invasive method of reducing pain during subcutaneous insulin injections in adults with type 2 diabetes mellitus. It is seen from these results that the local vibration technique used in different invasive interventions was effective in pain management, and that they are similar to the results of our study. It has been stated that the control of the vibration technique on pain is explained by gate control theory, according to which a stimulus such as the vibration technique activates the A-beta nerve fibers, closing the pain gates [23,33].

On the other hand, it has been concluded in some other studies that vibration during vaccination injections of children did not have a positive effect on the pain of vaccination [21,34]. Also, in a study evaluating the effect of different pharmacological methods in peripheral intravenous catheterization, it was concluded that the vibration technique did not have significant effect on the intensity of perceived pain during catheterization [26]. It was seen that the results of this study were different from those of the present study. It is thought that this difference may arise from such characteristics as the age of the participants included and the type of invasive procedure used.

Vibration devices have also been employed for non-cosmetic medical procedures in both adult and pediatric demographics, and evidence suggests that they represent a safe and effective approach for enhancing patient comfort [25]. In the present study, the Buzzy^®^ device was used to provide vibration. This device provides a combination of cold application and vibration, with ice packets providing the cold therapy and a motor in the body providing the vibration [30]. In the present study, only the vibration characteristic of the body of the Buzzy^®^ device was used. The reason for this was that because the absence of studies investigating the effect of vibration alone in the administration of subcutaneous LMWH injection was being considered, only the effect of vibration in the procedure was examined. It is also reported that the Buzzy^®^ device is effective in pain management through physical and cognitive-behavioral nonpharmacological methods [22,35]. With regard to nonpharmacological effects in pain management, the combination of cold application and vibration shows the physical effect, and attracting the attention elsewhere shows the cognitive-behavioral effect [35]. The technique used in pain control of directing the attention elsewhere is a nonpharmacological method based on the belief that an individual can be consciously aware of only one stimulus at a time [34]. In this method, all of the patient’s senses are engaged to focus his/her attention on other stimuli [36]. With this in mind, a placebo group was formed in this study because the Buzzy^®^ device has the characteristic of diverting the attention. The aim here was to assess whether vibration alone had an effect in subcutaneous LMWH injection. As a result of the study, it was concluded that placing a Buzzy^®^ device on the procedure area of the participants with the vibration inactive did not have as much effect as local vibration physically applied to the injection site. From this result, it can be said that the physical nonpharmacological method of vibration alone has a positive effect in the control of the pain of subcutaneous LMWH injection.

It is reported in the literature that with age, sensory mechanisms, behavioral, hormonal, and social factors may affect the perception of pain [37]. Also, it has been emphasized that there is probably a multi-directional relation between potential pain perception and the variables of gender [38], and BMI [39]. It was seen in this study that the variables of the participants’ age, gender, and BMI did not significantly affect the levels of the pain of subcutaneous LMWH injection. Different results have been seen in other studies. In some studies, the variables of age [26,40], gender [27], and BMI [41] have been found to be significantly correlated with pain severity, while in others, it has been concluded that the variables of age [22,32], gender [12,22,26,32], and BMI [22,29] have no significant effect on pain. The study results may have differed according to the participants’ individual variables, their pain tolerance characteristics, and the type of intervention.

### 4.1 Strengths and limitations

The strong aspects of the study were that it was a randomized, placebo-controlled, single-blind study, and the first clinical study to evaluate the effect of vibration in the administration of subcutaneous LMWH injections. However, it also had a number of limitations. The first was that the results cannot be generalized because the research was conducted at a single center and in a single clinic. In order to increase reliability in the reduction in the pain of subcutaneous LMWH injection, there is a need for studies with larger sample dimensions. A second limitation of the research is that only the evaluators did not know the application performed on the patient, and for this reason it was a single-blinded study. It is therefore recommended that performing double-blinded studies would increase the strength of the study. Another limitation of the study was that the pain sensitivity, needle phobia, and hemodynamic variables of the individuals who took part in the research, which could have affected pain severity, were not evaluated. There is a need for comparative studies to research these factors.

## 5 Conclusion

It was found that compared with the placebo and control groups, local vibration given to the injection region had the effect of reducing the pain of subcutaneous LMWH injection. It was also found that the variables of age, gender, and BMI had no effect on the severity of the pain of subcutaneous LMWH injection. The conclusion of the study showed that the application of local vibration provided by the Buzzy^®^ device during subcutaneous LMWH injections was an effective method in reducing patients’ pain. This result may help in the pain management of patients, and may have a positive effect in increasing their comfort and quality of life. The local vibration technique can be used by health professionals, especially nurses, in clinical applications. Also, the Buzzy^®^ device may be preferred by health institutions for its ease of use and reusability, and for its low cost.

## Abbreviations

BMI: body mass index
LMWH: low molecular weight heparin
VAS: visual analog scale
